# Machine Learning Approach for Metabolic Syndrome Diagnosis Using Explainable Data-Augmentation-Based Classification

**DOI:** 10.3390/diagnostics12123117

**Published:** 2022-12-10

**Authors:** Mohammed G. Sghaireen, Yazan Al-Smadi, Ahmad Al-Qerem, Kumar Chandan Srivastava, Kiran Kumar Ganji, Mohammad Khursheed Alam, Shadi Nashwan, Yousef Khader

**Affiliations:** 1 Department of Prosthetic Dentistry, College of Dentistry, Jouf University, Sakaka 72345, Saudi Arabia; 2 Department of Computer Science, Faculty of Information Technology, Zarqa University, Zarqa 13110, Jordan; 3 Department of Oral Maxillofacial Surgery & Diagnostic Sciences, College of Dentistry, Jouf University, Sakaka 72345, Saudi Arabia; 4 Department of Preventive Dentistry, College of Dentistry, Jouf University, Sakaka 72345, Saudi Arabia; 5 Department of Computer Science, College of Computer and Information Sciences, Jouf University, Sakaka 72345, Saudi Arabia; 6 Department of Public Health, Jordan University of Science & Technology, Irbid 22110, Jordan

**Keywords:** metabolic syndrome, data augmentation, feature selection, diagnostic algorithms, disease diagnosis

## Abstract

Metabolic syndrome (MetS) is a cluster of risk factors including hypertension, hyperglycemia, dyslipidemia, and abdominal obesity. Metabolism-related risk factors include diabetes and heart disease. MetS is also linked to numerous cancers and chronic kidney disease. All of these variables raise medical costs. Developing a prediction model that can quickly identify persons at high risk of MetS and offer them a treatment plan is crucial. Early prediction of metabolic syndrome will highly impact the quality of life of patients as it gives them a chance for making a change to the bad habit and preventing a serious illness in the future. In this paper, we aimed to assess the performance of various algorithms of machine learning in order to decrease the cost of predictive diagnoses of metabolic syndrome. We employed ten machine learning algorithms along with different metaheuristics for feature selection. Moreover, we examined the effects of data augmentation in the prediction accuracy. The statistics show that the augmentation of data after applying feature selection on the data highly improves the performance of the classifiers.

## 1. Introduction

Major and dramatic changes in the development of nations, economies, cultures, and the environment, as well as an enhancement of the standard of living, have resulted from breakthroughs in science and technology throughout the past century. However, these technological advances have had unintended consequences, including shifts and disruptions in people’s daily routines, the natural world, cultural practices, and societal and economic structures. Because of this, the population is more susceptible to various internal and external risk factors, any one of which may in turn generate pathological states that may ultimately lead to diseases. Infection with pathogenic bacteria, free radicals, carcinogens, toxic chemicals, pollutants, and genetic abnormalities are only some of the many risk factors that might contribute to the onset of these conditions [1]. Changes in way of life, nutrition, and exercise, as well as inactivity, have all been linked to the rise of metabolic illnesses [2]. The aforementioned risk factors may have contributed to the development of diseases such as metabolic syndrome, cardiovascular disorders, diabetes mellitus, cerebrovascular diseases, food-borne diseases, infectious diseases, and cancer [3]. Therefore, focusing on health indicators is an attractive opportunity to explore the health state of individual and population subjects in relation to biochemical changes in the body.

**Figure 1:**
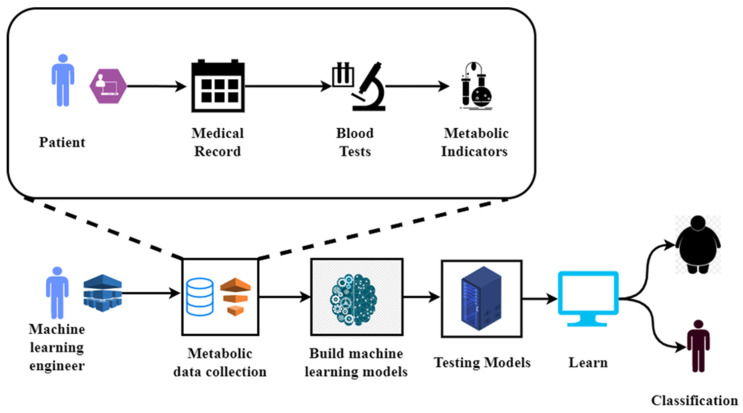
Metabolic syndrome diagnosis using machine learning.

Central obesity, high blood pressure, high blood sugar, and abnormal lipid profiles are the four main components of metabolic syndrome (MetS) [4]. It is important to note that rapid economic growth, an aging population, changes in lifestyle, and obesity are all contributing to the rising prevalence of MetS. The global prevalence of MetS is estimated to be between 20 and 25% [5]. MetS has been linked to an elevated danger of developing diabetes, heart disease, cancer, and mortality [6]. MetS poses a growing clinical and public health burden all over the world [7]. That is why it is crucial to implement effective measures to prevent and manage the spread of MetS. Data mining of medical checkup data can help identify patients at high risk of MetS at an early stage, advancing the timing of disease prevention and control from the later stages of disease development to the earlier stages of disease development. Preventing and controlling MetS requires the development of risk prediction models using data from physical examinations. Models for predicting the likelihood of a disease occurring are called disease risk prediction models [8]. These models are developed to identify those at high risk for a certain disease so that preventative or early intervention measures can be taken. Therefore, it is of considerable practical importance to build a MetS risk prediction model so that at-risk individuals can be identified and treated as soon as possible. Factors such as sex, age, and family history [9], as well as modifiable factors such as diet, physical activity level, and blood pressure [10] contribute to the onset and progression of MetS [11]. Modifiable risk factors are those that can, in theory, be altered. The best way to prevent and manage metabolic syndrome is to identify and address the underlying causes of the condition. However, identifying risk factors for MetS is complicated by interactions between risk factors [12]. In order to successfully deal with complex interactions between variables, machine learning, which is algorithm-based data analysis technology, is equipped with potent data analysis skills. These considerations led to the development of a machine-learning-based risk prediction model for MetS in this study. Figure 1 illustrates the metabolic syndrome diagnosis using machine learning [13].

In this paper, to establish a simple and practical risk prediction model for MetS, we built different machine learning models, based on easily available indicators such as demographic characteristics, anthropometric indicators, living habits, and family history of the subjects and then used a SHAP tool to explain and visualize the model. The interpretable MetS risk prediction model can help uncover risk factors, identify high-risk individuals, and provide methodological references for the prevention and control of MetS.

The key contributions are as follows: (1) Comparing the performance of numerous ancient and new statistical, bagging, and boosting machine learning models; (2) using the synthetic minority oversampling technique (SMOTE) as a data-augmentation strategy to solve the problem of unbalanced classes and avoid bias in machine learning models; (3) using multiple metaheuristics algorithms for feature selection to highlight the metabolic indicators with the best discriminative potential for machine learning models; (4) following feature selection, the Shapley additive explanations (SHAP) method is used to analyze machine learning model results and highlight the most significant metabolic indicators.

The rest of the paper is organized as follows: Section 2 focuses on relevant studies in the field. Section 3 illustrates the proposed methodology, data gathered, classifiers used, and metaheuristics methods, whereas Section 4 shows the statistical measures used for model assessment as well as the hyperparameter tuning process. Moreover, Section 5 discusses the experimental findings and SHAP’s explanations of machine learning models. Lastly, Section 6 concludes the study and suggests next directions.

## 2. Research Background and Related Works

In the field of metabolic syndrome data prediction and classification, a lot of new study methods, including experimental, investigative, empirical, and comparative research techniques, have been developed lately. Today, because of the availability of a large number of data sets that detail a variety of medical examinations, medical information, as well as the symptoms and indicators of particular diseases, has become an aid in the process of predicting diseases in their early stages. Metabolic syndrome is a prime example of this. The authors of [14] proposed a novel framework for the classification of metabolic data. This new framework makes use of random forests, C 4.5 classifiers, and JRip classifiers. They used a dataset that included information on the lifestyles and blood tests of 2942 patients living in Mexico City. In addition, Chi-squared was used as a feature selection approach in order to exclude irrelevant aspects of the data. According to the results, triglycerides, HDL cholesterol, and waist circumference are some of the greatest indicators of whether or not someone has metabolic syndrome. Regardless of the circumstances, the best results were obtained from random forests. The study in [15] utilizes clinical and genetic data from a Korean community that does not have an excessively overweight population. A framework was created with the use of the WEKA tool to simplify the employment of five different machine learning classifiers. These classifiers are known as naive Bayes, random forests, decision trees, neural networks, and support vector machines. They used data from a total of 10,349 different people. A few examples of the clinical variables that are discussed in the dataset are triglyceride levels, high-density lipoprotein cholesterol levels, and alcohol intake. In comparison to the other approaches, naive Bayes was successful in obtaining the largest area under the curve (0.69).

Using deep learning: In [16], a study of metabolic syndrome scores was proposed, and as part of that research, a dataset was offered that described the health of 3577 students in Birjand. The levels of glucose and triglycerides in the blood taken first thing in the morning are two blood tests that may be used as indicators. The distribution of the data, on the other hand, mandated the use of the synthetic minority oversampling technique for the purpose of achieving data parity. A linear discriminant analysis model was used in the process of feature extraction. The CART classifier outperforms neural networks and support vector machines in terms of four statistical metrics; however, these other two methods are more common. The prediction models, on the other hand, came to the conclusion that the most discriminating factors are waist circumference and high-density lipoprotein. In [17], a dataset consisting of 67,730 Chinese patients who had had a medical examination was used to evaluate the performance of random forest, XGBoost, and stacking classifiers. There were 32 predictors of physical medical tests, blood tests, and ages included in the information that was acquired. However, by employing cross-validation with 10 folds and the area under the curve metric, according to the statistics, XGBoost had the most area under the curve (93%) of any algorithm. In addition, the shapely additive explanation (SHAP) was used in the data to determine the relevance of the attributes. The fasting triglyceride level, abdominal obesity, and body mass index were shown to be the most significant indicators by the SHAP analysis for metabolic data prediction.

The study in [18] evaluates a dataset that describes medical information and tests for 17,182 patients using six of the most effective machine learning classifiers, including logistic regression, extreme gradient boosting, K-nearest neighbors, light gradient boosting, decision trees, and linear analysis. However, three statistical criteria were utilized to evaluate the models. Light gradient boosting exceeded the others, with an area under the curve of 86%. Furthermore, SHAP analysis revealed that waist circumference, triglycerides, and HDL-cholesterol had the strongest discriminative power for predicting metabolic data. In [19], decision trees and support vector machines were tested on an Isfahan cohort study dataset. The dataset described 16 metabolic syndrome indicators. However, for data balance, the synthetic minority oversampling technique was used. The WEKA tool and three statistical metrics were used in the experiment. According to the results, the support vector machine outperforms decision trees by 75% in terms of accuracy.

Using one-dimensional neural networks and eight additional machine learning classifiers: A study was conducted in [20] to evaluate a dataset that describes the lifestyles, medical information, and blood tests of 1991 middle-aged Korean patients. The dataset described several metabolic syndrome indicators such as age, smoking status, sleep time, and waist circumference. However, SMOTE was utilized for data balance. In addition, an area under the curve measure was utilized to evaluate the models. When compared to others, the findings demonstrate that XGBoost has the greatest performance, with an AUC of 85%. Furthermore, waist-to-hip ratio and BMI were shown to be the most important indicators for metabolic prediction. The study in [21] collected a dataset of 39,134 Chinese metabolic syndrome patients. The data set contained information on 19 different diagnostic tests, including alkaline phosphatase, prior diabetes, uric acid, and eosinophil percentage. The developed framework, on the other hand, assessed the employment of logistic regression, random forest, and extreme gradient boosting classifiers. Recursive feature elimination was also employed to select features. With an accuracy of 99.7%, XGBoost surpassed the others. In addition, the LIME library was utilized to display the significance of features. Fasting triglycerides, central adiposity, and systolic blood pressure were shown to be the most significant indications.

Using gradient boosted trees and logistic regression: In [22], an experimental study was conducted to investigate the usage of a Japanese metabolic syndrome dataset. The health insurance union data covered certain patients’ demographics and medical examinations, such as high-density lipoprotein cholesterol, anemia, and smoking. However, the proposed machine learning classifiers were fine-tuned. The area under the curve measure was also employed to evaluate the models. The findings, on the other hand, show that gradient-boosted trees performed best, with an AUC of 89.4%. Furthermore, other metabolic indicators, such as diastolic and systolic blood pressure, were shown to have no impact. The study in [23] was carried out in order to investigate the use of metabolic data from 5646 patients in Bangkok. On the other hand, in order to evaluate how well the random forest classifier worked, four different statistical methods were used. Additionally, ten-fold cross-validation and principal component analysis were carried out on the data. The research indicates that random forest had the best accuracy, coming in at 98.11%. Furthermore, it was shown that the triglyceride level was the single most important feature.

Table 1 summarizes the related research in terms of data gathered, machine learning classifiers employed, and key metabolic syndrome indicators. However, in comparison to prior contributions, in this paper, we compare the performance of ten statistical, boosting, and bagging machine learning classifiers on a metabolic syndrome dataset that includes 29 separate diagnostic procedures and medical tests. We also utilize five metaheuristic algorithms for feature selection, as well as SMOTE for data balance. Furthermore, we apply the Shapley additive explanations tool to explain the outputs of machine learning models at different data observation samples.

## 3. Proposed Metabolic Classification Framework

**Figure 2:**
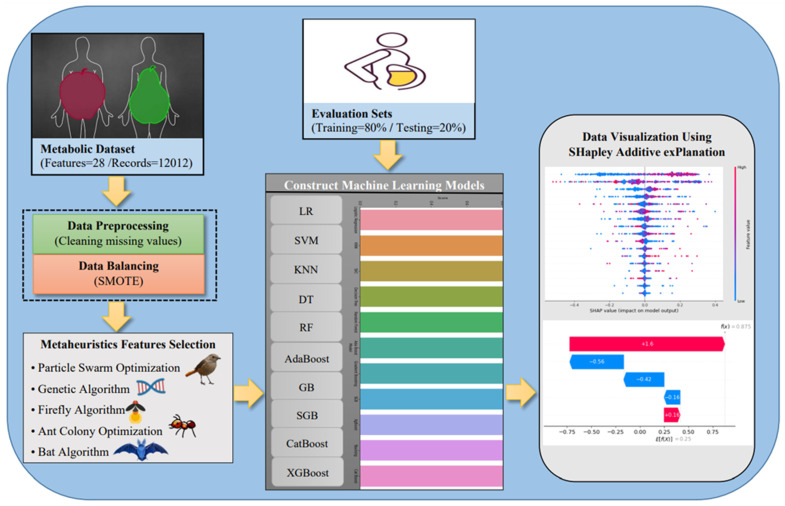
An overview of the proposed framework for metabolic data classification.

In the following sections, we illustrate the proposed framework. In this paper, we propose a new framework for metabolic data classification as shown in Figure 2. The designed framework evaluates the use of ten distinct machine learning classifiers such as logistic regression (LR), support vector machine (SVM), K-nearest neighbors (KNNs), decision trees (DTs), random forest (RFs), adaptive boosting (AdaBoost), gradient boosting (GB), stochastic gradient boosting (SGB), categorical boosting (CatBoost), and extreme gradient boosting (XGBoost) over a metabolic dataset obtained from the Kaggle repository. The dataset contains 29 distinct features that describe several patients’ statuses with a total of 12,012 records. Data preprocessing, on the other hand, was performed to clean the missing values (e.g., null values). Additionally, we noted that the metabolic dataset had a large target classes distribution that indicated imbalanced classes. Therefore, we followed the use of the synthetic minority oversampling technique (SMOTE) as a data resampling technique to balance the data classes. In this study, we evaluate the use of five distinct nature-inspired algorithms for feature selection such as particle swarm optimization, genetic algorithm, firefly algorithm, ant colony optimization, and bat algorithm. Finally, we employ the use of shapely additive explanation (SHAP) to explain machine learning models’ outcomes. SHAP [24] is an open-source python library that is a game-theory-based tool that visualizes how much a single or a group of observations adds to the predictive models by calculating the SHAP value; hence, based on the average predicted values from each classifier, it can be used to highlight and rank the most important features. The SHAP value can be computed as follows:

$$V=E\left[f\left(x\right)\right]=E\left[f\left(x\right)-f\left(x\right)\right]\;$$

where *E* represents the mean of predicted values of $f\left(x\right)$.

For evaluating machine learning classifiers, 20% of the data was maintained as a testing set. Our goal is to build accurate classification models for MetS analysis. The proposed framework has the potential to identify high-risk patients and give methodological references for MetS prevention and treatment.

### 3.1. Metabolic Data Collection and Analysis

Researchers have employed a variety of machine learning metabolic datasets over many years. In this paper, we follow the use of a metabolic dataset that is available at the Kaggle repository. The dataset includes details on 28 different diagnostic procedures, patient demographics, and indicators of metabolic syndrome. The features of the dataset are summarized in Table 2. Nonetheless, we recorded that out of a total of 348,348 data samples (data total population) in the dataset, 23.301 had at least one missing value. However, missing values were cleaned via data preprocessing. In this study, we follow the use of dropping all data records with missing values. In addition, the dataset was free of outliers. In this work, we construct ten machine learning models with different meta-heuristics algorithms for data evaluation and classification.

### 3.2. Synthetic Minority Oversampling Technique for Data Balancing

Due to the presence of unbalanced data, often referred to as imbalanced target classes, the classifiers that are used by machine learning systems may be biased in favor of one category over another. On the other hand, there is a significant disparity in the distribution of target classes among metabolic datasets. Because of this, we made use of synthetic minority oversampling technique (SMOTE) to guarantee that every piece of data was dispersed fairly. SMOTE [25] develops synthetic data samples to increase minority class data samples by first locating the K nearest neighbors, then calculating the distance between those neighbors, and then increasing that distance by a random integer that falls between 0 and 1.

### 3.3. Machine Learning Classifiers

#### 3.3.1. Logistic Regression

The objective of a logistic regression model is the same as that of a linear regression model, which is to make predictions about the dependent variable by examining the relationship between the independent variables. However, rather than making continuous predictions, it may be used to answer questions with yes or no. Given that $h_{\theta }\left(x\right)=1/(1+e^{-\theta^{T}}\cdot x)$, the sigmoid function is used to fit an s-shaped line with a predicted value between 0 and 1. This allows for the possibility that y = 1, which is correct given the value of x. In addition, the sigmoid function is used to fit an s-shaped line with a predicted value between 0 and 1 [26]. When it is predicted that Y will be 0.5, a value of 0.5 for x indicates that there is a probability of 50% that Y will be 1. A low x number suggests a low likelihood that y = 1, whereas a high x value indicates a high probability that y = 1, and an intermediate x value indicates a 50% chance that y will be 1.

#### 3.3.2. K-Nearest Neighbors

The K-nearest neighbors (KNNs) classifier [27] is a comparison method that is based on the distance between the two sets of data. Because it keeps data samples all through the learning phase and then learns throughout the assessment phase, the KNNs classifier is an algorithm that is known for its slowness. When KNNs is seated, it goes through a variety of different stages. We first determine the value of K, which is the number of samples that are the closest to the new data sample, and then apply a distance function to determine how much the new data sample deviates from the training samples. The majority of class values are utilized to determine the prediction value. Based on the value of K, the distance values are sorted, and the samples that are physically nearest to each other are chosen.

#### 3.3.3. Decision Trees

DTs [28] are a classifier that group the characteristics that are present in the training data based on the judgments that are made by trees. Predictions in DTs are made by drawing conclusions in response to a collection of questions that have a significant influence on the intended result of the prediction. These inferences are based on the answers that individuals provide to the questions. This classifier is referred to as a non-parametric approach since it does not need the use of a mapping function to link predictors and outcomes. The term “impurity” is used in DTs to refer to the procedure of determining the decision values that are optimal for each depth level. On the other hand, the function known as the Gini index may be used to determine how impure the tree is.

#### 3.3.4. Random Forests

For classification, RFs [29] use a bagging ensemble. They learn the basics by creating many decision trees from the training data. Similarly, RFs are not a parametric model. To create classification output, however, just a small random sample of rows is selected from the whole data set, and a set of decision trees is constructed for each subset. Decision trees may vary in size. A meta-classifier is a choice on the classification that is made using the basic learners’ judgments as input, and it is indicated by a majority vote in RFs. They can handle simple and complex predictor functions, as well as numeric and categorical data.

#### 3.3.5. Support Vector Machine

The number of features that are input into a support vector machine help to identify the appropriate decision boundary in high-dimensional space. The classifier is the one who uses this boundary after it has been determined. The purpose of the support vector machine, given that there is a wide variety of hyperplanes from which to choose, is to select the hyperplane that maximizes margins over all of the data samples [30]. In addition, the classification may be carried out either in a linear or non-linear way. When the class distribution is known, support vector machines often perform better and are less likely to succumb to the problem of overfitting. On the other hand, if one makes use of the biggest margins available, it is feasible that new data may be correctly fitted and categorized.

#### 3.3.6. Adaptive Boosting

An ensemble of machines is used in the classification method known as AdaBoost [31], which is a boosting classifier. Unlike random forests and decision trees, this kind of forest is ordered. When using AdaBoost, a number of decision trees are joined in a manner that is decentralized. Each one of them is referred to as a “stump,” and it is made up of a single node in addition to two leaves. A forest of stumps is a collection of tree trunks that have been chopped down to their bases and left standing together. A stump, on the other hand, is not a very good learner when it comes to learning how to classify things. The purpose of AdaBoost is to combine numerous weak learners for classification to work together on a classification problem. Because the output of one tree may influence the output of the next, the AdaBoost algorithm places a significant amount of weight on the order in which stumps are generated. When it comes time to rank the stumps, each data sample is assigned a weight (w) that is proportional to the total number of samples as a whole. The formula $sw=w_{old}\times e^{amount\;of\;say\;\left(\alpha \right)}$ is used on a regular basis in order to make adjustments to the sample weight. The Gini index is another method that may be used to evaluate the relevance of a stump; typically, a lower Gini score indicates more significance.

#### 3.3.7. Gradient Boosting

GB [32] is a method for classification and regression that is based on boosting ensembles and makes use of pseudo residuals (PR). Gradient boosting differs from AdaBoost in that it does not begin with a stump, but rather with a single leaf that reflects the median values of the predicted classes. AdaBoost begins with a stump. In contrast to that, GB creates a tree of a certain size, very similar to how AdaBoost performs it, with the key difference being that each tree may be far more substantial than a stump. In GB, decision trees serve as the base learners. The probability ratio is the loss function that may be determined by contrasting the actual values with the projected values. This strategy is a powerful one, since it allows prediction errors in GB to be reduced to a minimum via the consistent updating of PR values across trees.

#### 3.3.8. Stochastic Gradient Boosting

Friedman first described the method that uses an ensemble of learners and is known as stochastic gradient boosting [33]. The homogenous hybrid approach, which involves boosting and bagging, was the impetus behind it. The process of weighing the base learners may be used to perform both regression and classification. On the other hand, the SGB base learners with the highest adoption are decision trees. On the other hand, in order to train each tree, a randomized selection of records from the data sample is employed. SGB is a potent method for lowering the chance of overfitting via the random elimination of a sample of input data. This is accomplished when compared to bagging procedures, which are not as effective. SGB is used throughout a wide range of areas, and this practice dates back many decades.

#### 3.3.9. Extreme Gradient Boosting

XGBoost [34] is one of the ensemble methods that make use of boosting. Other ensemble methods include adaptive and gradient boosting. This new technology has replaced the older technique known as gradient boosting. In the same vein as its predecessor, it was intended to handle enormous and complicated data sets. In contrast to adaptive and gradient boosting, XGBoost makes use of innovative regression trees in its algorithm, changing into what is now referred to as reborn trees. However, in contrast to gradient boosting, XGBoost trees begin with a single leaf rather than several leaves. In addition, the regularization and gradient boosting steps of XGBoost are not extras that may be skipped. XGBoost has been one of the most successful boosters on the market for a significant amount of time. The discriminative accuracy of its predictions is quite high, and it is not difficult to put into practice. In addition to this, it has the capability of managing large datasets with imbalanced target classes.

#### 3.3.10. Categorical Boosting

CatBoost [35] is an improvement on gradient boosting. In 2018, it was delivered by Anna Veronika Dorogush and the rest of the Yandex business team. It was conceived with the intention of being preferable to bagging and stacking for a number of different kinds of data. It is based on a method known as boosting, and it was designed to handle information of both category and numerical kinds. CatBoost is a free software package that enables highly rapid calculations on central processing units (CPUs) as well as graphics processing units (GPUs). It is very effective for usage with relatively small datasets despite its ease of implementation. In addition to this, it is a technique that is based on decision trees, which helps avoid overfitting. CatBoost has been in a head-to-head battle with a variety of gradient boosting algorithms during the course of its existence.

### 3.4. Metaheuristics Features Selection

#### 3.4.1. Genetic Algorithm (GA)

Utilizing a genetic algorithm, which is a population-based meta-heuristic, is one method that may be used to address optimization problems [36]. The organization of chromosomes had a significant impact on the development of the algorithm, which may be thought of as a kind of search. The following is a list of the steps involved in GA: Establish the population by means of its fitness function, subsequent selection, and subsequent reproduction. Gene parameters are first allocated to individuals whose genomes have been randomly created before being utilized to produce chromosomes. The idea that each individual has something to offer to the process of finding a solution to a problem is one of the core principles behind GA. Instead, the fitness function is used to assign a numerical value to each individual, which indicates whether or not they are fit for reproduction. This value might be positive or negative. After the choosing process is complete, the crossover function could be used to create a new individual as the next step.

#### 3.4.2. Ant Colony Optimization (ACO)

To this day, ACO remains one of the most popular meta-heuristic search-based algorithms, used in a broad variety of fields. This new method is a refinement of the ant system published by Marco Dorigo in 1992. ACO [37] was conceptualized by seeing how ant colonies go about their foraging. The goal of ant colony optimization is to find the quickest route from the anthill to the food source. Each path represents a potential answer. However, after the paths have been established, ants use organic chemical molecules called pheromones to guide them along the most direct route to their nest. ACO has been used to and improved upon a wide variety of models.

#### 3.4.3. Particle Swarm Optimization (PSO)

The PSO optimization approach obtains its inspiration from the behaviors of birds that are found in their natural environments [38]. Within the context of this population-based research approach, each individual bird is referred to as a particle, while the whole flock is referred to as a swarm (i.e., population). In PSO, each “particle” is effectively the ideal response to the problem that is being addressed. The positions of the particles, however, will be rearranged once the first randomization has been performed. PSO has been used widely in medical data classification. In [39], PSO was used for metabolic syndrome risk quantification.

#### 3.4.4. Firefly Algorithm (FA)

The use of FA, a kind of meta-heuristic method, may be used for a wide range of optimization problems. It is an algorithm based on natural phenomena, namely the flashing behavior of fireflies. The firefly algorithm is founded on the idea that fireflies are attracted to one another as a swarm, with the less dazzling ones following the brighter ones [40]. This is because the brighter fireflies attract the attention of the other fireflies. Each firefly represents a potential answer, and the closer that firefly goes to the optimal solution, the brighter it grows, which encourages other fireflies to follow in its footsteps. FA has been used often in medical data collection for a great many years. The authors of [41] used FA in order to enhance the classification capabilities of the AdaBoost algorithm for liver diseases.

#### 3.4.5. Bat Algorithm (BA)

The BA optimizer is a meta-heuristic approach that may be used to solve optimization problems. It is an algorithm that draws its inspiration from nature and is based on the way microbats use echolocation [42]. A type of bat known as the micro bat makes use of echolocation, which may be thought of as a kind of sonar, to help it locate prey. The idea behind echolocation sonar is that little bats emit a loud sound in the shape of a wave, and the wave is reflected back to the microbats by the prey it is directed towards. The process of selecting features to classify medical data has traditionally made extensive use of BA. In the study [43], the bat algorithm was altered to accommodate the classification of breast cancer data.

## 4. Evaluation Metrics

Some statistical evaluation metrics, such as the area under the curve, precision, recall, root mean squared error as prediction error function, and receiver operating characteristic curve, can be used to determine the degree of relationship between the performance of machine learning models and the amount of data. In this work, we evaluate ten different machine learning classifiers by employing different metrics of precision, recall, area under the curve (AUC), and testing accuracy that are based on a confusion matrix, as shown in Equations (1)–(4). Despite the fact that these metrics show the performance of classifiers in terms of successfully classified data and data that was mistakenly categorized, in order to enhance the accuracy of the classifiers, we made use of the GridSearchCV method to determine the optimal values for the boosting-based classifiers’ hyperparameters [44]. As can be seen in Table 3, we choose how many estimators to use and how many different values for the learning rate to include when defining the grid search space. In addition, in order to conduct the experiment, we formulated two distinct loss algorithms and functions along with five-fold cross-validation. The following are the four key basic elements of the confusion matrix:

- **True positive (TP):** Presents the number of infected patients that have been classified correctly as infected patients.
- **True negative (TN):** Presents the number of non-infected patients that have been classified correctly as non-infected patients.
- **False positive (FP):** Presents the number of misclassified non-infected patients that are infected.
- **False negative (FN):** Presents the number of misclassified infected patients that are non-infected patients.

(1) $$Accuracy=\;\frac{TP+TN}{TP+FP+TN+FN}\;\;\;\;$$

(2) $$Precision=\frac{TP}{TP+FP}\;$$

(3) $$Recall=\frac{TP}{TP+FN}\;$$

(4) $$AUC=True\;positive\;rate\;\left(Sensitivity\right)=\frac{TP}{TP+FN}$$

$$\;False\;positive\;rate=1-specificity=1-\frac{FP}{TN+FP}$$

## 5. Metabolic Data Classification Experimental Results and Model Explanations

**Figure 3:**
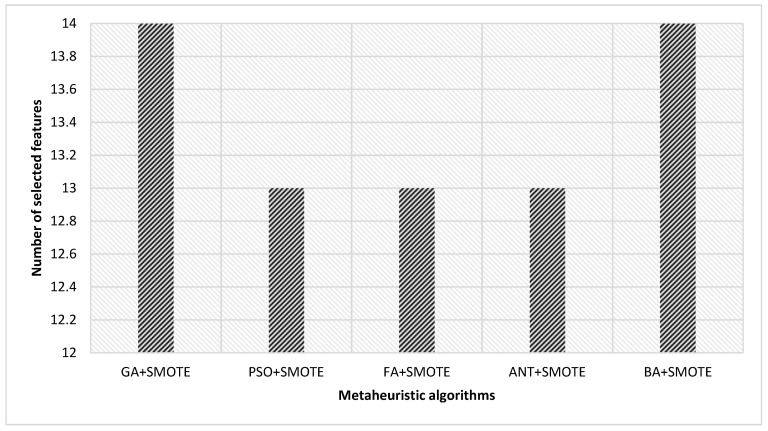
The total number of selected features utilizing metaheuristics algorithms.

The next section discusses the experimental findings that were obtained by using the framework that was devised. In this investigation, five different metaheuristics algorithms for feature selection were carried out. Table 4 demonstrates the fine-tuning that was performed on the proposed metaheuristic algorithms. There are a total of six hyperparameters that required adjustment: chaotic type (µ), number of iterations (β), heuristic rate (η), number of generations (α), crossover function probability (ϓ), and mutation probability (Ψ). The selected features have been shown to have the greatest discriminative power for prediction models that make use of metaheuristics methods. Figure 3 shows the total number of selected features utilizing metaheuristics. For clarity, we found that subject age, gene a, gene b, gene c, gene d, breathing rate, high blood pressure, high triglyceride, comprehensive metabolic panel test results, maternal pregnancy record, premature delivery, per MCL quantity of white blood cells, maternal abortion count, and reduced HDL were among the most informative features and indicators for metabolic syndrome based on metaheuristics performance.

The testing accuracy results of several classifiers that were evaluated on the metabolic syndrome dataset using metaheuristic approaches are shown in Table 5. The data, taken as a whole, show an increase in accuracy. Using the genetic and bat algorithms, however, the KNNs classifier outperforms others with a 94.4% accuracy rate. To be clear, this is due to the KNNs algorithm performing better with fewer features, and so the number of features was lowered following the feature selection process utilizing metaheuristics techniques. Another explanation is that KNNs is a distance-based and sluggish algorithm by nature, therefore it outperforms others in the evaluation stage owing to data distribution and training, which is not surprising. On the other hand, the performance of boosting ensemble-based classifiers was superior to that of statistics and bagging classifiers. It was found that the GB, SGB, and CatBoost classifiers had the highest improvement in results, with an average accuracy range increase of between 33% and 35%. This was established by comparing the new findings to the pure results. However, this is not surprising given the fact that boosting-based classifiers decrease bias by increasing variance. In contrast to boosting classifiers, the logistic regression classifier exhibited a 15% gain in accuracy. This is because logistic regression is a linear model, but metabolic data distribution has nonlinear decision boundaries. Additionally, in order to determine which metaheuristic algorithms provided the best results, the average testing accuracy was computed. Notably, the genetic algorithm and the bat optimizer were superior to other methods, since they had an average testing accuracy of 78.1%. In addition, it was discovered that the classifiers KNNs, DTs, GB, SGB, and CatBoost represented significant results when comparing precision and recall, as is shown in Table 6 and Table 7. However, by calculating the average value of precision and recall, it was found that particle swarm optimization, firefly algorithm, and ant colony optimizer had the highest outcome improvement, with an average value of 80% for precision and 79% for recall. Table 8 shows the findings of employing AUC to evaluate the effectiveness of the classifier. However, it is noteworthy that RFs, GB, SGB, and CatBoost classifiers exhibited the best performance, with an AUC of 96.9% as the highest result.

**Figure 4:**
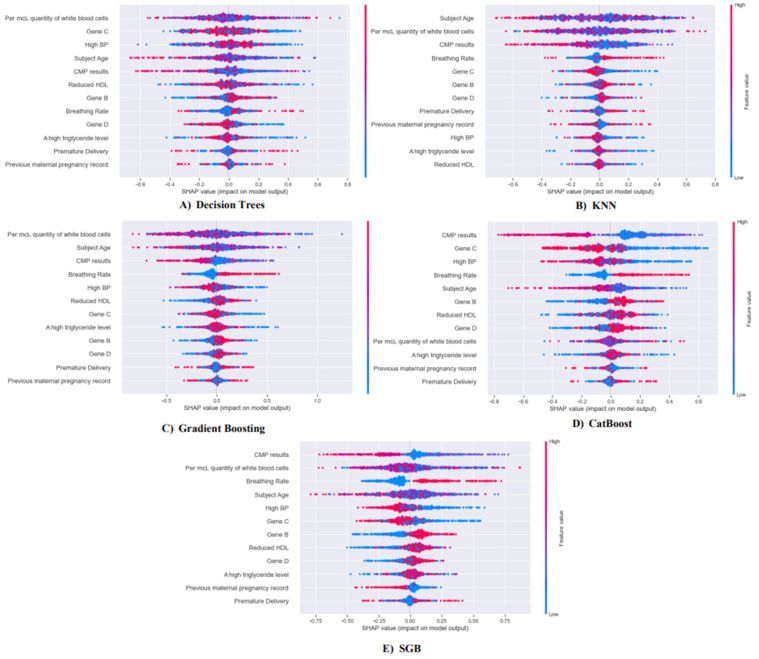
The most informative metabolic indicators using PSO, FA, and ANT optimizers. Blue dots represent a low contribution to the predictive model, whereas red dots represent high impact. (**A**) Shows quantity of white blood cells as the most important feature according to DTs. (**B**) Shows the subject age as the most important feature according to KNNs. (**C**) Shows quantity of white blood cells as the most important feature by GB. (**D**,**E**) Show the comprehensive metabolic panel test as the most informative feature according to CatBoost and SGB.

For the purpose of this study, we made use of the SHAP library to explain the results of machine learning models. On the other hand, the bee swarm plot was used to demonstrate the most discriminative metabolic syndrome indicators in comparison to the prediction models. Figure 4 shows the most informative features determined by DTs, KNNs, GB, CatBoost, and SGB with the use of the optimizers PSO, FA, and ANT. These classifiers demonstrated the most significant increase in terms of testing accuracy, precision, recall, and area under the curve (AUC). Nonetheless, we found that the comprehensive metabolic panel test, the quantity of MCL white blood cells, the subject’s age, high blood pressure, and breathing rate were the most important metabolic indicators via SHAP.

Furthermore, when comparing the importance ranking of features from one classifier to another, it is worth noting that these rankings may differ depending on the feature selection methods utilized and the process of tweaking their hyperparameters. Nonetheless, in this study, we investigated the application of SMOTE as a data-augmentation strategy that minimizes the sensitivity of classifiers to new data samples. As a result, our goal was to build accurate classification models that are as stable as possible. However, another restriction is that we highlighted other significant variables that might give sufficient and effective metabolic indicators, such as body mass index and waist circumference, that did not exist in the dataset employed.

**Figure 5:**
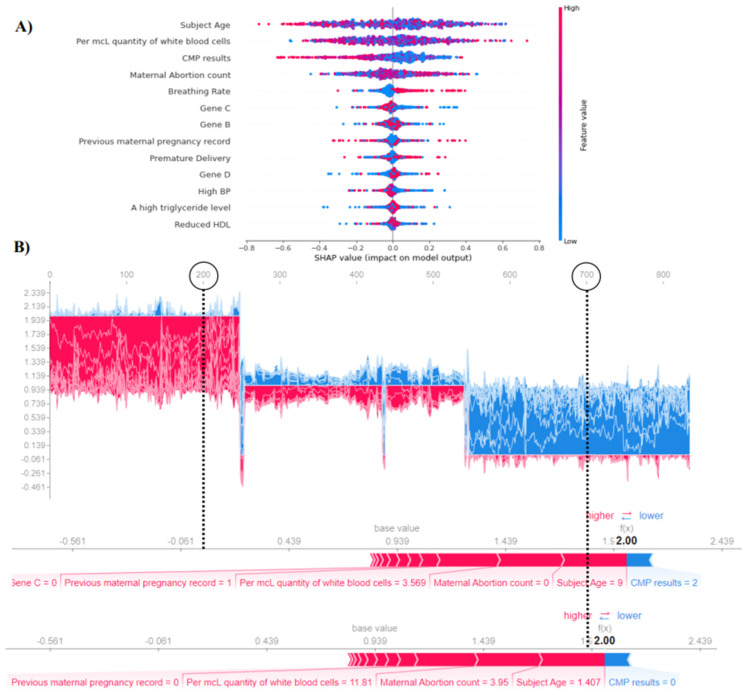
SHAP analysis over the KNNs classifier utilizing GA and BA optimizers. (**A**) Shows the most informative metabolic indicators found via KNNs. Blue dots show negative impact, whereas red dots show positive impact. (**B**) Shows SHAP global and local force plots at data observations of 200 and 700.

Figure 5 shows a SHAP analysis of KNNs performance that was accomplished using genetic and bat optimizers. KNNs achieved a score of 94.4% accuracy throughout testing, which was higher than any other classifier. Nevertheless, in order to explain the results of the model, we make use of two different SHAP plots: the global force plot, which shows model outcomes over a variety of data observations, and the local force plot, which illustrates model outcomes across a single data observation. In spite of this, we chose two observations from the data at random, 200 and 700, to investigate which metabolic indicators are the most significant. Subject age of 9, maternal abortion count, per MCL quantity of white blood cells of 3.569, and previous maternal pregnancy record indicators have a positive impact and contribution to the KNNs classifier, according to the SHAP analysis of KNNs, which was based on the SHAP base value of 0.939. On the other hand, the KNNs classifier was used in the bee swarm plot, which presents an illustration of the most important features overall. The variables that were found to have the highest feature values were age, the quantity of white blood cells per MCL, a comprehensive metabolic panel, the number of maternal abortions, and the breathing rate.

**Figure 6:**
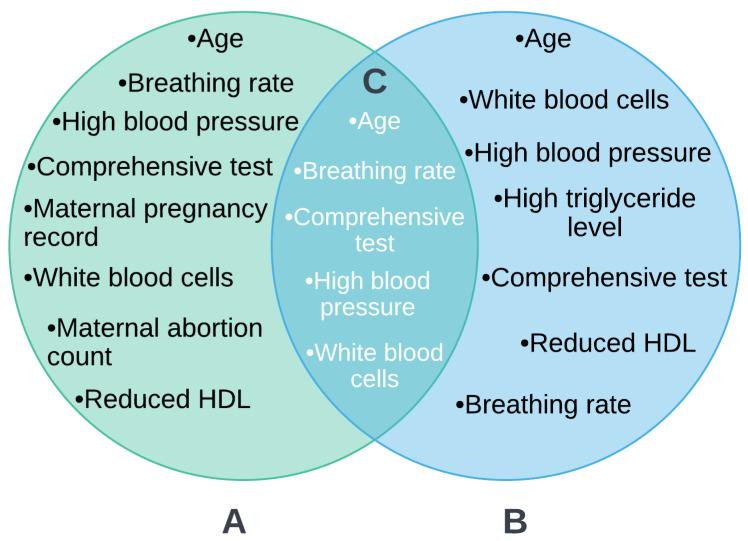
An analysis of the intersection of the most important metabolic indicators found via metaheuristics algorithms and SHAP. (**A**) Shows a set of common selected features by ACO, GA, PSO, FA, and BA algorithms as the most important indicators. (**B**) Shows as set of classified features as the highest discriminative power for prediction by SHAP values. (**C**) Shows an intersection for the final most informative features between metaheuristic algorithms and SHAP.

To go further for medical considerations, first we filtered metabolic indicators using a straightforward, methodical technique that consisted of two main stages: choosing features using metaheuristics algorithms and then interpreting them using the SHAP tool. Figure 6 shows the intersection of the most significant common features selected using five metaheuristic algorithms and a set of the most important features rated as having the highest discriminating power using SHAP using a Venn diagram. However, based on common features selected by the suggested approaches, such as age, hypertension, white blood cell count, comprehensive medical tests, and raised respiratory rate, it can be inferred that machine learning models obtain the highest accuracy in prediction and classification. However, medical considerations must be taken into account to explain why these qualities were chosen. Therefore, we aim in this study to take medical considerations into account.

For medical considerations, we discuss the ranking of top-importance features as determined by SHAP values below. It should also be noted that the top five most important metabolic indicators are the patient’s age, comprehensive metabolic panel blood tests, per MCL quantity of white blood cells, breathing rate, and high blood pressure. However, when it comes to patient age, we found that the patient’s risk of developing metabolic syndrome increases with age. Seven years of research published in [45] found that the likelihood of having metabolic syndrome has increased since the 1990s. People above the age of 50 were more likely to be obese than those younger. It is also worth mentioning that as people become older, some disorders emerge that have a high likelihood of producing metabolic syndromes, such as insulin resistance, the development and advent of heart disease, and vascular diseases. These disorders, which worsen with age, have been linked to the development of metabolic syndrome [46]. Therefore, it may be argued that age plays a significant influence in predicting whether or not an individual is impacted by metabolic disease. However, when it comes to comprehensive metabolic panel blood testing, we found that it covers glucose, a type of sugar that the body needs for energy, and that a high glucose rate may indicate a risk of developing metabolic disease. It also includes measures for carbon dioxide, potassium, chloride, triglyceride levels, and cholesterol. Comprehensive metabolic blood tests have identified these as the most common metabolic syndrome criteria [47]. Furthermore, triglyceride and cholesterol levels were defined as features in the utilized dataset. As a result, based on data distribution, they provide a strong indicator of the significance of the whole metabolic blood test characteristic. As a result, it was evaluated as the first and third most important indication according to the majority of classifiers.

Furthermore, we found that due to their influence on the body’s immunity, white blood cells are one of the first lines of defense in the body. As a result, the higher the number of white blood cells, the stronger the body’s resistance to diseases, and vice versa. Nonetheless, the studies in [48,49] emphasize a relationship between white blood cells and metabolic syndrome. Additionally, they noticed a correlation between white blood cells and other tests, such as high blood pressure, which was identified as another crucial metabolic indication using machine learning algorithms in this study. Some other measurements related to the number of white blood cells are insulin, triglycerides, and body mass index. Therefore, it can be concluded that people with metabolic syndrome have more white blood cells, in addition to some observations on the high levels of some proteins such as C-reactive [50]. The metabolic syndrome, on the other hand, is heavily influenced by breathing rate. Increasing the rate of breathing raises the body’s metabolism, which enhances the body’s ability to burn extra fat and vice versa.

For high blood pressure, which is also known as hypertension, it was found by prior studies that there is a strong correlation between blood pressure and metabolic syndrome [51], particularly in the case of severe hypertension. It is stressed, however, that increasing body mass and weight causes a rise in blood pressure. It is also correlated to other tests such as heart pulse rate, where an overweight person’s heart pulse rate rises to allow blood to circulate to the body, which raises blood pressure. However, it should be noted that some studies found that the relationship between high blood pressure and metabolism is still not fully understood, and therefore they found that the body’s insulin resistance and visceral obesity scale are classified as risks leading to high blood pressure in metabolic syndrome [52]. As a result, machine learning algorithms recognized high blood pressure as one of the most significant metabolic indicators, owing to its strong association with other data such as pulse rate.

To summarize and highlight the proposed methodology’s state of the art in comparison to prior methods, the goal of this work is to highlight the highest indices of discriminative metabolism by interpreting machine learning models using the SHAP approach. Other research revealed significant metabolite indicators that must be considered when developing effective prediction models using feature selection approaches. However, this is not enough to explain all predicted values generated using machine learning models. Therefore, by obtaining the average predictive values for the collection of predicted indicators and evaluating the performance of classifiers at certain samples, we may achieve a reasonable degree of interpretation to understand the reasons behind machine learning models’ preference for some features over others. Furthermore, earlier research has concentrated on the application of a variety of specialized types of machine learning models. However, in this study, we investigated and compared the application of ten machine learning classifiers from all classes, including statistical, bagging, and boosting, to build and find the most accurate models for data classification.

Moreover, when compared to prior studies, we applied data augmentation approaches such as SMOTE to lower the degree of sensitivity of machine learning models to future data. Additionally, it has the advantage of not copying data records when compared to other data resampling methods; instead, it generates synthetic data samples, in addition to the examination of the use of natural-derived algorithms such as metaheuristics, which is regarded as one of the contemporary ways of selecting attributes when compared to other methods such as filter and wrappers techniques. However, we noted that the majority of prior research concentrated on the usage of certain data quality in a given region and culture. As a result, the goal of this study was to employ a dataset that focuses on metabolism at the public level without regard to culture or geography.

## 6. Conclusions

Metabolic syndrome is not a condition that stands on its own as a disease. Instead, it is a collection of risk factors, including high blood pressure, high blood sugar, abnormal cholesterol levels, and fat accumulation in the abdominal region. In this paper, we propose a new framework for classifying metabolic syndrome data. The framework designed has the ability to identify high-risk individuals and provide references for MetS prevention and therapy. Ten machine learning models of various statistical, bagging, and boosting types were evaluated in this study using different patients’ medical data, blood tests, four gene types, and metabolic panel indicators. Five metaheuristics algorithms were used for feature selection: particle swarm optimization, genetic algorithm, bat algorithm, firefly algorithm, and ant colony optimization. The main contribution of this paper, however, is to explain the outputs of machine learning models and highlights the most important metabolic indicators using the Shapley additive explanation library. In addition, we used SMOTE as a data resampling technique for data balance. The findings show that KNNs outperforms others, with a testing accuracy of 94.4% and AUC of 84.4%. Furthermore, we found that patient age, comprehensive metabolic panel blood test, per MCL quantity of white blood cells, breathing rate, and high blood pressure are among the top five most informative metabolic indicators when compared to machine learning models. Additionally, we concluded that GA and BA surpass others, with an average testing accuracy of 78.1%.

## Figures and Tables

**Table 1:** Summary of the related works.

Paper	Dataset	Classifiers	Metaheuristics	Metabolic Best Indicators	Results
[14]	2942 patients living in Mexico City	RFs, C 4.5, JRip	NO	Triglycerides HDL cholesterol Waist circumference	RFs outperforms others.
[15]	10,349 of non-obese Korean patients	NB, RFs, DTs, ANN, SVM	NO	NO	NB best performing with AUC (69%).
[16]	3577 students in Birjand	CART, ANN, SVM	NO	Waist circumference High-density lipoprotein	CART classifiers outperform others.
[17]	67,730 Chinese patients	RFs, XGBoost, stacking	NO	Fasting triglyceride level Body mass index Abdominal obesity	XGBoost best results with AUC (93%).
[18]	Medical tests of 17,182 patients	LG, XGBoost, KNNs, LGB, DTs, linear analysis	NO	Waist circumference Triglycerides HDL-cholesterol	LGB best results with AUC (86%).
[19]	Isfahan cohort study dataset	SVM, DTs	NO	NO	SVM best results with an accuracy of 75%.
[20]	Medical tests of 1991 middle-aged Korean patients	1D NN, DTs, NB, KNNs, XGBoost, RFs, LG, SVM, ANN	NO	Waist-to-hip ratio BMI	XGBoost best results with an AUC (85%).
[21]	39,134 Chinese metabolic syndrome patients	LG, RFs, XGBoost	NO	Fasting triglycerides Central adiposity Systolic blood pressure	XGBoost with an accuracy of 99.7%.
[22]	Japanese metabolic syndrome dataset	GBT, RFs, LG	NO	NO	GBT performs the best with an AUC of 89.4%
[23]	Medical tests of 5646 patients in Bangkok	RFs	NO	Triglyceride level	RFs with an accuracy of 98.11%.

**Table 2:** Metabolic dataset description and analysis.

Metabolic Dataset Description	
Features	Description
**Subject ID**	Describes the patient’s ID.
**Subject age**	Describes the patient’s age.
**Gene A**	Describes the patient’s Gene A in DNA.
**Gene B**	Describes the patient’s Gene B in DNA.
**Gene C**	Describes the patient’s Gene C in DNA.
**Gene D**	Describes the patient’s Gene D in DNA.
**Per MCL quantity of blood cells**	Describes the patient’s blood cells per microliter. The typical range of adults is between 4.35 to 5.65 million blood cells.
**Breathing rate**	Describes the patient’s breathing rate. It is a measurement to check if the patient has breathing difficulty.
**Pulse rate**	Describes the patient’s heart pulse rate.
**Diagnostic testing**	Describes if the patient has any medical test records.
**Carrier testing**	Describes if the patient has had any carrier test (a type of genetic test that is used to determine if the patient is a carrier of specific diseases) before.
**Enzyme test**	Describes if the patient has had any enzyme test (a blood test that measures if the patient has a specific disease) before.
**Insulin test**	Describes if the patient has any insulin test records.
**Thyroid test**	Describes if the patient has records of any thyroid tests (a type of blood test that is used to measure thyroid performance).
**Gender**	Describes the patient’s gender (male/female).
**Gastrin defect**	Describes if the patient has a gastrin hormone defect or not.
**Neural anomaly**	Describes if the patient has any neural anomaly tests.
**Presence of severe allergies**	Describes if the patient has any allergies.
**Premature delivery**	Describes if the patient has any premature delivery record (indicates an early baby birth).
**Assistance needed in fertility**	Describes if the patient has needed any assistance in fertility.
**Previous maternal pregnancy record**	Describes if the patient has any previous maternal pregnancy record.
**Maternal abortion count**	Describes the patient’s total number of abortions.
**Per MCL quantity of white blood cells**	Describes the patient’s white blood cells per microliter.
**CMP results**	Stands for the comprehensive metabolic panel, which is a blood test that provides information about body metabolism.
**High triglyceride level**	Describes if the patient has high triglyceride.
**Reduced HDL**	Describes if the patient has a low cholesterol level, which indicates a potential for heart disease.
**High BP**	Describes if the patient has high blood pressure.
**Metabolic syndrome type**	Target classes.
**Total number of features**	28
**Total number of records**	12,012
**Total number of data in population**	348,348

**Table 3:** GridSearchCV search space values for classifier hyperparameter tuning.

Parameters	Grid-Search Space
Number of estimators	50, 70, 90, 100, 120, 150, 180, 200
Learning rate	0.001, 0.01, 0.1, 1, 10
Loss function	Deviance, exponential
Algorithms	SAMME, SAMME.R

**Table 4:** Metaheuristics algorithms for feature selection hyperparameter tuning.

Meta-Heuristics Algorithms	Hyperparameters	Population Size
ACO	µ = logistic map, β = 20, η = 0.7	20
GA	α = 20, ϓ = 0.6, ψ = 0.033	20
PSO	β = 20, ψ = 0.01	20
FA	µ = logistic map, ψ = 0.01, β = 20	20
BA	µ = logistic map, β = 20, ψ = 0.01	20

**Table 5:** Classifier testing accuracy results over metabolic dataset utilizing metaheuristics.

Classifiers	Pure	(PSO/FA/ANT)	(GA/BAT)
Logistic regression	0.51727	0.669	0.6746
KNNs	0.4623	0.930	0.9449
SVM	0.5110	0.724	0.7236
DTs	0.4469	0.821	0.8169
RFs	0.5110	0.660	0.6578
AdaBoost	0.51	0.66	0.6578
GB	0.5285	0.879	0.8887
SGB	0.5276	0.845	0.8588
CatBoost	0.5326	0.7954	0.8349
XGBoost	0.5126	0.661	0.6495
Accuracy (AVG)	0.5074	0.755	0.7814

**Table 6:** Classifier performance using precision metric.

Classifiers	(PSO/FA/ANT)	(GA/BAT)
Logistic regression	0.66	0.67
KNNs	0.93	0.94
SVM	0.72	0.72
DTs	0.83	0.82
RFs	0.68	0.71
AdaBoost	0.82	0.71
GB	0.88	0.89
SGB	0.84	0.86
CatBoost	0.79	0.84
XGBoost	0.67	0.66
Precision (AVG)	0.8	0.79

**Table 7:** Classifier performance using recall metric.

Classifiers	(PSO/FA/ANT)	(GA/BAT)
Logistic regression	0.67	0.67
KNNs	0.93	0.94
SVM	0.72	0.72
DTs	0.82	0.82
RFs	0.66	0.67
AdaBoost	0.82	0.67
GB	0.88	0.89
SGB	0.84	0.86
CatBoost	0.80	0.83
XGBoost	0.67	0.66
Recall (AVG)	0.79	0.78

**Table 8:** Classifier AUC results.

Classifiers	(PSO/FA/ANT)	(GA/BAT)
Logistic regression	0.837	0.838
KNNs	0.868	0.844
SVM	0.839	0.852
DTs	0.802	0.797
RFs	0.947	0.953
AdaBoost	0.761	0.758
GB	0.969	0.959
SGB	0.969	0.963
CatBoost	0.941	0.967
XGBoost	0.896	0.857
AUC (AVG)	0.888	0.883

## Data Availability

Not applicable.
